# Use of Smartphone-Based Head-Mounted Display Devices to View a Three-Dimensional Dissection Model in a Virtual Reality Environment: Pilot Questionnaire Study

**DOI:** 10.2196/11921

**Published:** 2019-01-14

**Authors:** Yoshihito Masuoka, Hiroyuki Morikawa, Takashi Kawai, Toshio Nakagohri

**Affiliations:** 1 Department of Surgery Tokai University School of Medicine Kanagawa Japan; 2 Faculty of Science and Engineering Waseda University Tokyo Japan

**Keywords:** medical education, smartphone, virtual reality

## Abstract

**Background:**

Virtual reality (VR) technology has started to gain attention as a form of surgical support in medical settings. Likewise, the widespread use of smartphones has resulted in the development of various medical applications; for example, Google Cardboard, which can be used to build simple head-mounted displays (HMDs). However, because of the absence of observed and reported outcomes of the use of three-dimensional (3D) organ models in relevant environments, we have yet to determine the effects of or issues with the use of such VR technology.

**Objective:**

The aim of this paper was to study the issues that arise while observing a 3D model of an organ that is created based on an actual surgical case through the use of a smartphone-based simple HMD. Upon completion, we evaluated and gathered feedback on the performance and usability of the simple observation environment we had created.

**Methods:**

We downloaded our data to a smartphone (Galaxy S6; Samsung, Seoul, Korea) and created a simple HMD system using Google Cardboard (Google). A total of 17 medical students performed 2 experiments: an observation conducted by a single observer and another one carried out by multiple observers using a simple HMD. Afterward, they assessed the results by responding to a questionnaire survey.

**Results:**

We received a largely favorable response in the evaluation of the dissection model, but also a low score because of visually induced motion sickness and eye fatigue. In an introspective report on simultaneous observations made by multiple observers, positive opinions indicated clear image quality and shared understanding, but displeasure caused by visually induced motion sickness, eye fatigue, and hardware problems was also expressed.

**Conclusions:**

We established a simple system that enables multiple persons to observe a 3D model. Although the observation conducted by multiple observers was successful, problems likely arose because of poor smartphone performance. Therefore, smartphone performance improvement may be a key factor in establishing a low-cost and user-friendly 3D observation environment.

## Introduction

### A Virtual Reality and Three-Dimensional Model

In light of its recent growth and development, virtual reality (VR) technology has been gaining attention as a new system for potential introduction in education and training environments and as a form of surgical support in medical settings [1-5]. An increasing number of three-dimensional (3D) textbooks, such as the *3D Dissection Atlas* series, are being studied and read to test their usefulness [6,7].

Owing to tools such as the OsiriX DICOM Viewer (Pixmeo) and the SYNAPSE VINCENT volume analyzer (Fujifilm), it is now easy to build 3D models based on image data taken from patients’ actual cases [8,9]. Thus, expectations that 3D constructed models will become a form of surgical support are growing [10,11]. Furthermore, 3D models are useful for surgical teams in terms of image sharing. Presenting cases preoperatively using 3D models and visualizing actual previous surgeries provide immense positive outcomes as well as major educational benefits [12]. There are also numerous reports showing attempts at using VR technology in the process of surgery planning and/or navigation in the area of hepato-biliary-pancreatic surgery [2,13].

There are currently many types of 3D-modeling software tools, each equipped with distinctive features. How users employ the models differs depending on the needs of each user; whether or not users find the performance of these models satisfactory also differs accordingly.

### Smartphone

Older cell phone types have been replaced by the now ubiquitous smartphones, and we have recently entered an era in which everyone owns at least one of these extremely useful and convenient devices. Many advanced functions of these smartphones are being considered for their potential and/or availability for use in actual medical settings [14,15]. Smartphone and tablet apps for educational use have been developed and are becoming more available. Smartphone apps concerned with health care and medicine include digital books (eg, textbooks and guidelines) as well as sensors and video functions. Development of such medical apps that handle symptom evaluation, education, and rehabilitation has also been reported [16,17]. Reports on using smartphone video functions have recently increased [18,19], showing that high-definition smartphone cameras have also improved. Smartphones and their linked apps have enabled the use of VR and/or augmented reality (AR) environments through lenses using simple activation.

### Head-Mounted Display

Various head-mounted display (HMD) devices, such as Oculus (Facebook), VIVE (HTC), and Hololens (Microsoft), have been developed and are available in the market today. Moreover, wearable devices such as Google Glass (Google) and Hololens [10,20-23], the usefulness and feasibility of which are being studied, are used for medical purposes. 3D model presentation methods include both monitors and HMDs; nowadays, 3D printers are also employed [24,25]. We believe that when 3D models are used in medicine or medical science, the method or environment in which the models are observed will differ according to the costs in terms of time and economics, considering the extra time and cost it would require to prepare several numbers of the same HMD devices and/or install them so that they link and make the same movements.

In harnessing HMD for multiple persons to observe the same model, the number of HMD devices to be used will be the same as the number of observers. For this reason, it is costlier to teach and provide operating instructions to observers. On the contrary, as smartphones are now widespread, using them to share data and observe models could provide a simple, low-cost observation environment, which we consider highly feasible. However, we have yet to determine which system is the most practical to observe 3D models and identify problems that could arise when a new system of employing smartphone-based simple HMD devices is in practical use.

### Aim of This Study

This is the first study conducted for medical education purposes by using a smartphone-based HMD. It aimed to analyze potential issues of observing a 3D model of an organ that was produced based on an actual surgical case with a simple HMD using a smartphone. In addition, we evaluated and gathered feedback on the performance and usability of the simple observation environment that we created.

## Methods

### Flow of Experiment

A pilot study was conducted in the Department of Gastroenterological Surgery at Tokai University, where 17 medical students performed 2 experiments to observe 3D dissection models through a simple HMD. The targeted participants conducted the 3D model observations in 2 experiments: one by a single observer and another by multiple observers. Upon completion, they assessed the results by responding to a postexperimental questionnaire survey. To maintain consistency, we explained the details and flow of the experiment process to the participants before the experiments began. The following subsections describe the experiments.

### Participants

The participants consisted of 17 medical students at Tokai University who were in their fifth year of medical school and had studied anatomy. Tokai University’s clinical study ethical review board (17R112) reviewed and approved the study, and each participant provided written consent.

### Apparatus and Setting

**Figure 1:**
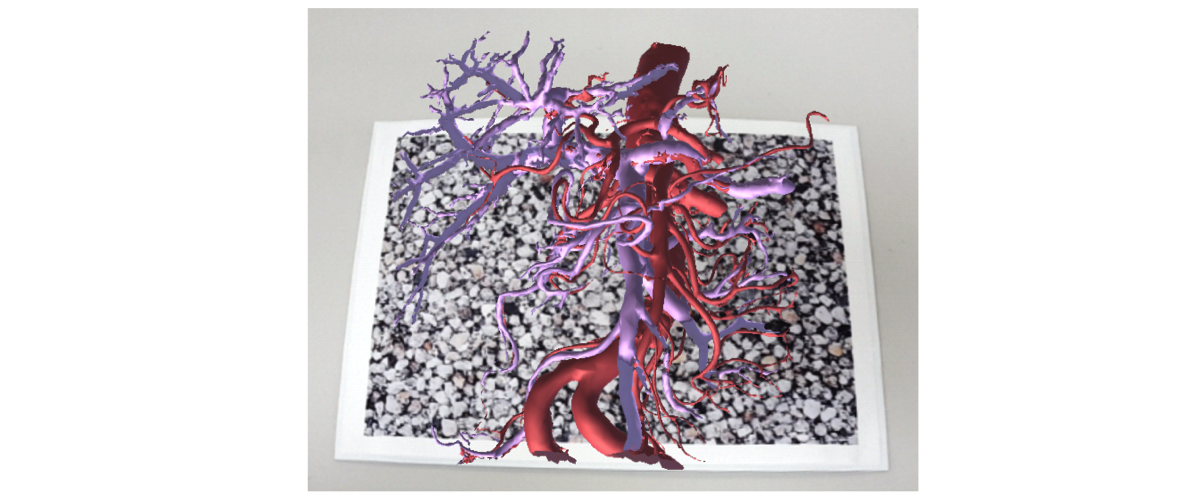
Three-dimensional (3D) dissection model. We performed a simple automatic extraction using 3D surface rendering of OsiriX and the modeled arteries (in red) and portal vein branches (in purple).

We performed a simple automatic extraction using 3D surface rendering by OsiriX (Pixmeo) and modeled arteries and portal vein branches (Figure 1). We used a smartphone (Galaxy S6; Samsung, Seoul, Korea) and downloaded the resulting data into it. We also used Unity (Unity Technologies) for displaying 3D models on smartphones.

**Figure 2:**
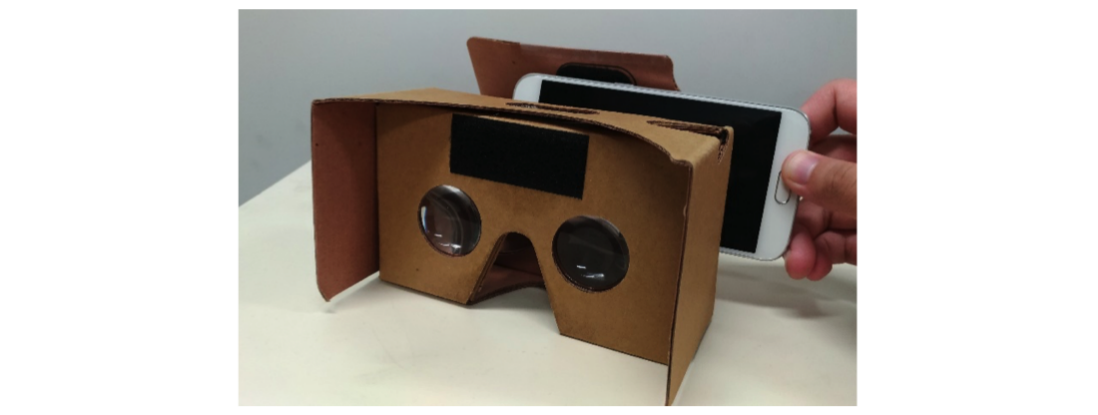
A simple head-mounted display (HMD) system. We built a simple HMD system using a smartphone (Galaxy S6 by Samsung) and Google Cardboard.

Next, we used Google Cardboard to create a simple HMD system (Figure 2). The Google Cardboard was created in compliance with the Google VR specifications [26]. The diameter of the lens was 34 mm, and the distance between the centers of both lenses was 64 mm. The actual measurement of the camera’s angle of view was 55 degrees and that of the HMD was 59 degrees. The distance between the lens and the virtual monitor was 667 mm, but the actual visual distance was 685 mm, as the length between the lens and the eye was 18 mm. In addition, the smartphone weighed 136 g and the cardboard 79 g, and the total weight of the HMD was 215 g.

**Figure 3:**
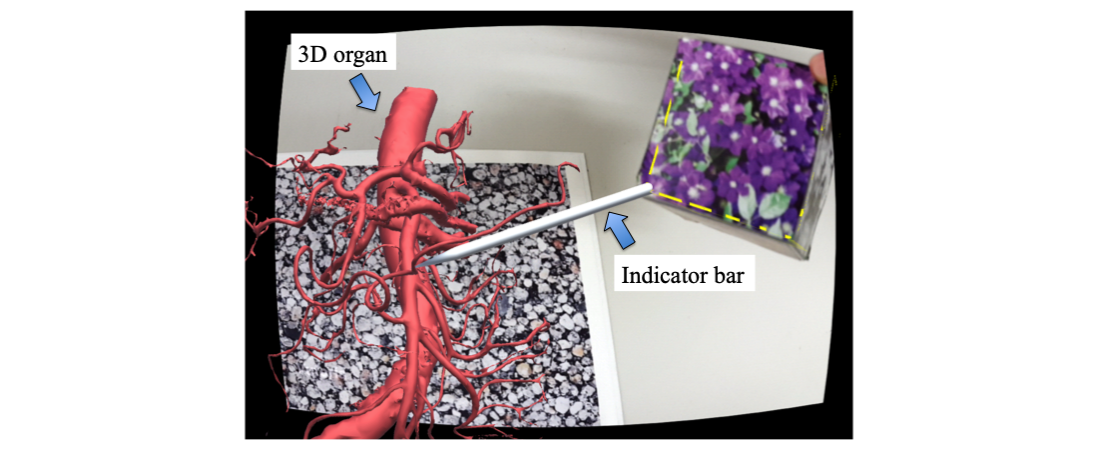
The participant’s perspective. When the smartphone’s camera recognized 2 augmented reality markers simultaneously, the three-dimensional organ from the sheet and indicator bar from the box appeared in front.

The system we built was capable of sharing a model between 2 HMD devices by applying AR markers (through the Vuforia platform; PTC). AR markers triggered the display of the virtual information. When we view AR markers through digital cameras based on image recognition technology, content that matches the digital camera image is displayed, appearing as if it is actually right in front of us. In this experiment, the AR markers consisted of 1 sheet and 1 box. When the device recognized them simultaneously, the 3D model from the sheet and the indicating bar from the box appeared on the display, which the participants were able to view (Figure 3). The size of the AR marker used on the sheet was 270 × 190 mm, whereas the AR marker used as an indicator bar was made from a cube (70 mm sides) and a paper drawing glued together. The indicator bar was designed to pop out from one corner. The length of the sharp bar was 100 mm. We chose natural images for the drawings (paper) used on each marker to make them recognizable.

This specification enables the observer to view the dissection model at a distance of 685 mm from the screen. At a magnification of 16.8 times, together with the smartphone screen width of 47 mm, the visual field of the virtual monitor will expand accordingly, with a 59.9-degree field of view. According to an actual observation, the 3D model and indicator bar were displayed on the screen without delay.

### Experimental Design and Data Collection

**Figure 4:**
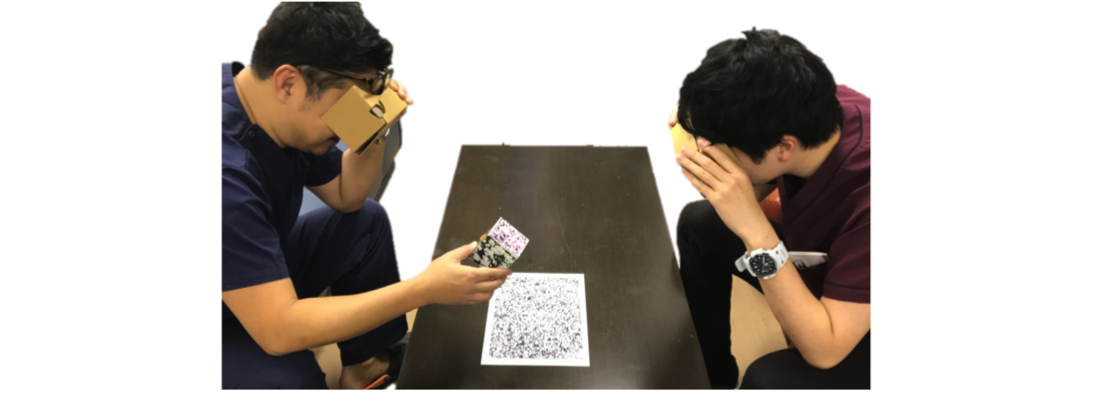
Participants’ observation of the three-dimensional (3D) model using the head-mounted display (HMD) system. In Experiment 2, participants faced each other and were asked to observe the 3D model through the simple HMD system.

We asked the participants to observe the 3D model through the simple HMD system (Figure 4) and evaluate the results by responding to a questionnaire survey.

#### Experiment 1: Observation by a Single Observer

Participants observed 3D models using an HMD device while reading a text on anatomy. Even if they had the HMD attached, they were able to see the text through the smartphone’s camera. They performed an observation exercise using this HMD to carry out the second experiment (Textbox 1). Afterward, they made an assessment using a 5-level Likert scale, ranging from *invisible* to *visible* (1-5, respectively).

#### Experiment 2: Multiperson Observation

In this experiment, participants paired up and took turns. One participant with the *box* marker indicated an artery or vein, whereas the other answered our questions, as shown below (Table 1). After the experiment, they assessed the results on a 5-level Likert scale, ranging from *strongly disagree* to *strongly agree* (1-5, respectively). We then gathered their opinions and impressions and prepared an introspective report. The participants filled out a usability questionnaire on the system. We calculated the overall scores attained by all participants.

#### Statistical Analysis

The items of the scored questionnaire were analyzed through Pearson correlation analysis using SPSS for Windows, version 18.0 (IBM Japan).

**Dissection name (abbreviation shown in brackets) used for blood vessels confirmation (Experiment 1). We chose the blood vessel titles shown in the model. The participants confirmed each blood vessel while reading a textbook. Afterward, the participants performed an assessment using a 5-level Likert scale, ranging from invisible to visible (1-5, respectively).**

- Common hepatic artery (CHA)
- Left gastric artery (LGA)
- Splenic artery (SpA)
- Portal vein (PV)
- Superior mesenteric vein (SMV)
- Inferior mesenteric vein (IMV)
- Splenic vein (SpV)
- Gastroduodenal artery (GDA)
- Right gastric artery (RGA)
- Proper hepatic artery (PHA)
- Right and left hepatic artery (Right/left HA)
- Anterior superior pancreaticoduodenal artery (ASPDA)
- Inferior pancreaticoduodenal artery (IPDA)
- Superior mesenteric artery (SMA)

**Table 1:** Experiment 2: usability of the head-mounted display (HMD) system. We assessed the usability of the HMD system. After the experiment, the participants marked the results on a 5-level Likert scale, ranging from *strongly disagree* to *strongly agree* (1-5, respectively).

Evaluation		Statement
**Visual image**		
	Image quality	The image quality was good enough
	Reality of the object	The reality of the object was good enough
**Device**		
	Size perception	The size perception was acceptable enough
	Distance perception	The distance perception was acceptable enough
**Usability of the wearable device**		
	Comfort	It was comfortable to use
	Heaviness	It was light to the touch
	Motion sickness	I did not feel sick from using it
	Eye fatigue	I did not experience eye fatigue from using it
	Total usability	This HMD had acceptable usability

## Results

### Experimental Results

**Figure 5:**
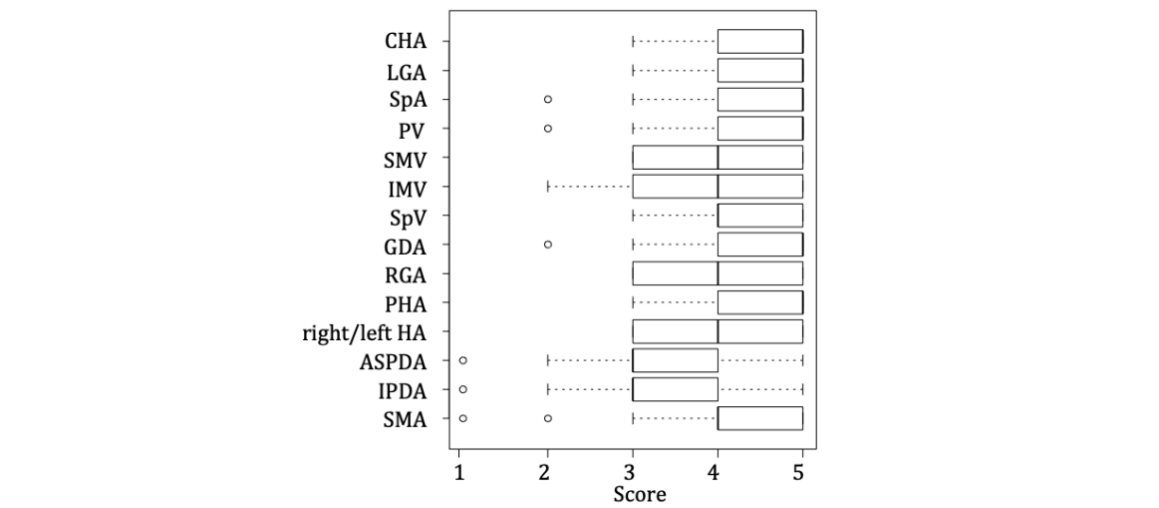
Dissection model assessment. The vertical axis indicates dissection names and the horizontal axis shows assessment scores. The results are as shown on a 5-level Likert scale, 5 points for “greatly understood” and 1 point for “did not understand.” Points for each organ are shown in the box plot. ASPDA: anterior superior pancreaticoduodenal artery; CHA: common hepatic artery; GDA: gastroduodenal artery; HA: hepatic artery; IMV: inferior mesenteric vein; IPDA: inferior pancreaticoduodenal artery; LGA: left gastric artery; PHA: proper hepatic artery; PV: portal vein; RGA: right gastric artery; SMA: superior mesenteric artery; SMV: superior mesenteric vein; SpA: splenic artery; SpV: splenic vein.

**Figure 6:**
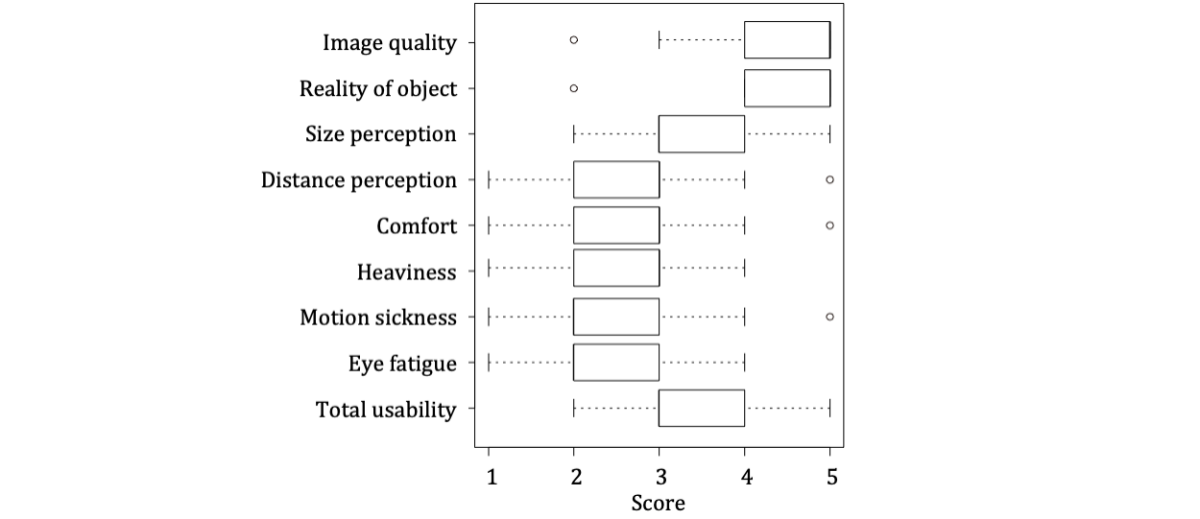
Usability assessment. The vertical axis shows assessment items. The horizontal axis indicates evaluation scores. The results are as shown on a 5-level Likert scale, 5 points for “very good” and 1 point for “very bad.” With respect to heaviness, motion sickness, and eye fatigue, the points are in inverse proportion to the burden. Each assessment item is shown in the box plot.

The assessment of the direction model yielded a largely favorable outcome (Figure 5). In terms of clear image quality, reality of object, size perception, and overall usability, the evaluation of the observation was high. In an introspective report on the observation experiment conducted by a single observer, more than half of the respondents responded that their spatial understanding improved compared with when reading a textbook. As for simultaneous observation by multiple observers, positive comments referenced the clear image quality and shared understanding. On the contrary, we received a low rating because of visually induced motion sickness and eye fatigue caused during the process (Figure 6).

### Introspective Report on Simultaneous Observation

**Figure 7:**
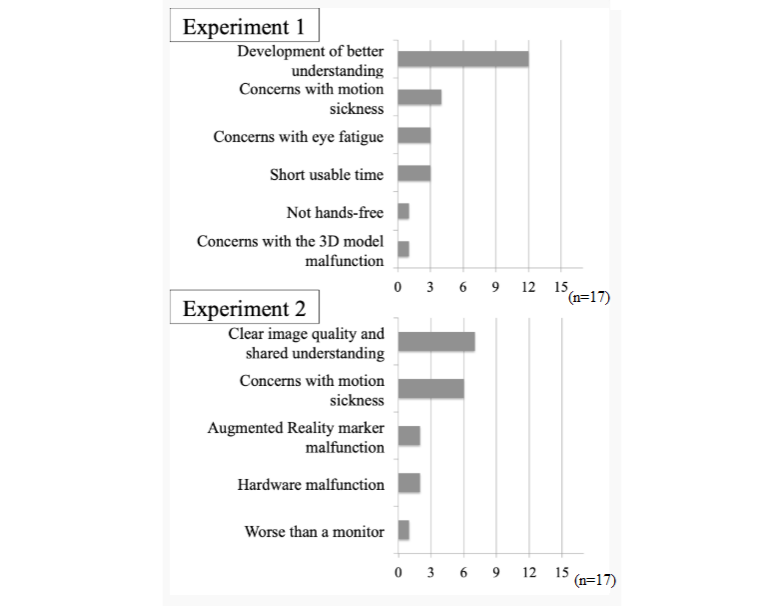
Introspective report on simultaneous observation. There were a total of 17 participants. The vertical axis indicates the comments received in the descending order of their frequency. The number of participants who provided the comments is shown on the horizontal axis (bar chart). 3D: three-dimensional.

Negative comments were also received because of hardware failure (specifically slow smartphone performance caused by heating issues and problems with AR markers; Figure 7). As a result of visually induced motion sickness during the experiment process, some respondents suggested that observation through a monitor would be a better choice.

## Discussion

### Simultaneous Observation

In this research, we were able to observe a 3D dissection model that had been extracted from patient data using a simple HMD. Smartphones, which are now widespread, are capable of observing a 3D model of a surgical patient by downloading the 3D model data. The moment such data are downloaded, this system becomes available to anyone with access to a smartphone, Google Cardboard, and the patient’s computed tomography (CT) data. This enables the user to hold a 3D surgical case conference anywhere. The 3D model here is a simple one created with the OsiriX viewer, and although issues remain in terms of smartphone performance, a detailed model is implementable. In addition, we believe that the most meaningful part of this experiment was that we were able to share the same model information with multiple observers in this observation environment, in which we used smartphone-based HMD devices.

We used Google Cardboard (created in compliance with the Google VR specifications) in the system. As the settings (eg, distance between both eyes and focal length) are fixed and there is no room for even a minor adjustment, it may help address the negative effects of VR sickness and/or eye fatigue by, for example, adjusting the lenses based on each individual. In sharing the 3D model information, we used AR markers instead of fingers to display the indicating bar. Thus, we were able to direct the dissection of the 3D model. Our use of the smartphone’s narrow angle of view may have also contributed to the restricted work space, making the recognition of the AR markers more difficult. Presumably, this can be avoided by using wide lenses. However, as the viewing angle is extremely narrow when compared with the Oculus Rift or HTC VIVE, a further comparative study is required. The total weight of the HMD is 215 g. It is relatively light as it is made of cardboard, but we need to keep holding it in our hands during its use. According to the introspection report, its light and user-friendly features received positive comments. On the contrary, negative comments were received regarding the burden of having to hold it every time. During the experiment, some participants had to hold down the smartphone with their hands to stop it from moving within the cardboard. Therefore, something that could keep the smartphone fixed on one’s head may be needed. Observation was the only task performed at this time, and participants’ feedback was rather favorable as the experiment did not require large movements, such as head adjustments. We look forward to the comments that we will receive when we add tasks other than observation in our future experiments.

**Figure 8:**
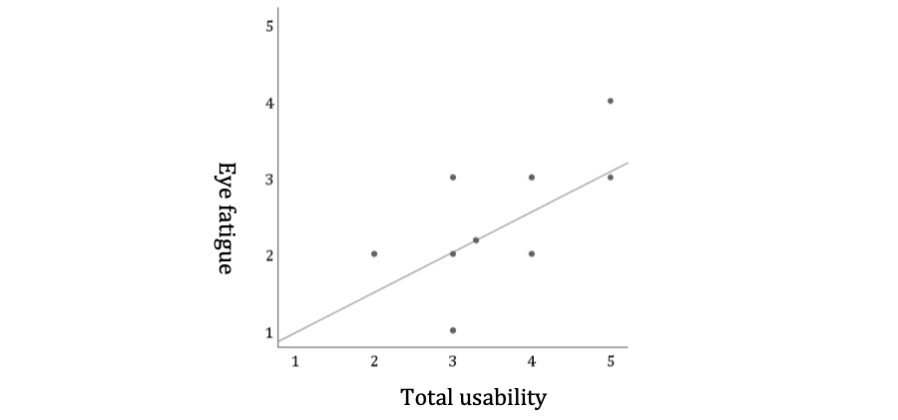
Correlation between eye fatigue and total usability. The vertical axis shows eye fatigue scores. The higher is the score, the less is the burden. The horizontal axis indicates the total usability scores. Eye fatigue showed close correlation with total usability (r=0.526, P=0.02).

### Virtual Reality Sickness

The presence of motion sickness, cyber sickness, and VR sickness along with various physiological symptoms was observed during the experiments; these are thought to be caused by parasympathetic activities and/or visual flow [27,28]. This seems to be related to various complex factors. Issues concerning the technical aspects of the VR environment (eg, HMD settings) are also observed. In previous studies where the Oculus Rift was used [29], motion sickness resulted in a VR environment and varied by gender. However, no gender difference was observed in this study. Although stereo vision is crucial in 3D depth perception and is considered advantageous [30] in terms of hand-eye coordination or driving technique, the prevalence rate of stereo blindness among the participants who lacked this vision was 1% to 30% [31]. It is thus possible that stereo blindness affected motion sickness or eye fatigue. In the case of observation conducted by multiple observers, displaying AR markers simultaneously resulted in smartphone heating, which eventually led to slow performance of the phone. Furthermore, adjusting head positions to display AR markers is likely to have caused motion sickness and eye fatigue. In fact, eye fatigue showed close correlation with total usability (*r*=.526, *P*=.02; Figure 8). Keeping score of VR sickness is suggested, and this suggestion is undergoing review [32]. To assess the issues of motion sickness, we need to keep a log of certain items (eg, general discomfort, fatigue, eyestrain, difficulty in focusing, headache, blurred vision, dizziness, and vertigo) to have further discussions.

### Simple System

In this research, we focused on how we can establish a simple system with ease, as well as on the prevalence of an environment in which medical students can learn or study preoperatively. The observers were not allowed to control the device except for moving (adjusting) viewpoints. The extent to which our HMD (using smartphones) can achieve this is still under discussion; however, we have started working on our model observation. Although this system, which can be created by using just a smartphone and a cardboard, is enough to perform a 3D model observation, it is not adequate for performing more complicated activities.

Our goal this time was to observe a simple HMD. Previous research that used the HMD reported that their aims included establishing a remote education system of surgical methods [23] and a system using the Oculus Rift to create a simulation or medical VR environment [33]. These studies are considered useful in clinical practice and/or surgery settings. Thus, from now on, we need to not only observe but also implement an interaction that could help perform tests (anatomy comprehensive exams) on training grounds with the use of our system as well as assess complex interactions, such as by implementing models (to be excised, etc), which appear exactly as they would in an actual surgery. Moreover, participants’ responses included requests for new functions such as a dissection title display feature for learners as well as on and off buttons to switch between each blood vessel model. However, in such a case, adding complex tasks (eg, transformation of 3D models in proportion to the surgery progress) may alter said evaluation. Furthermore, although it seems crucial to solve issues such as motion sickness, these problems may be solved naturally with the development of simple systems supported by the technological advancement of smartphones and AR markers.

In this study, we were not able to implement this system in an operating room or observe it in clinical practices because of ethical approval conditions. As we need to work in coordination with the hospital’s system to conduct a 3D observation on all patient data, it would be necessary to design an elaborate system. Currently, our simple system may be suited for case studies that present images of unique cases. As reflected by the results of Experiment 1, the evaluation of the 3D model quality was “agreeable.” As for the dissected parts with low evaluation, although the visual image may have been inadequate, it was enough to obtain and comprehend a rough image of the dissection. This point may also require assessment from a surgeon. Taking into account requirements from educational or clinical practices, we need to consider where this system will be needed or how we can develop this system in the future.

### Limitations

We currently face limitations such as hardware constraints (ie, system failures due to heating and/or recognition precision limits of the AR markers). To address this, we asked 5 surgeons from a hepato-biliary-pancreatic surgery group (Department of Gastroenterology, Tokai University) to conduct an observation and provide feedback by responding to some survey questions. The results were as follows: all 5 surgeons agreed on the clear image quality, and 3 of them had favorable reactions to the user-friendly device owing to its simplicity and compact size. Their positive comments reflected how the system enables intuitive observation from different angles as opposed to observation via monitors, making it easier to create a distinct image of the surgery (simulation), as the operator and his or her assistants usually stand face-to-face during the operation. However, nobody chose to use the 3D device over the two-dimensional (2D) CT test for preoperative checking. This is because of the limited information 3D models can provide compared with CT graphics (original data). To be specific, in addition to the vascular system’s graphics, images (such as those of tumors and other organs) are considered necessary as well. Thus, for detailed information, there is nothing more preferable to original data. We assume this to be the reason that the surgeons tend to choose 2D images over 3D models (shown on this system) for preoperative planning. Nevertheless, letting medical students or interns perform observations using 3D models should have positive educational effects. Some comments referred to the following possibility: if we install a function into the system that enables us to draw images onto the 3D space, it will enable us to conduct conferences with detailed information with surgeons who could draw additions or alterations onto rough 3D models while explaining and discussing them. Thus, we now need to bring the system to clinical sites and gather various types of requests. Future work in the field of surgery (eg, surgical conferences and education) will most definitely involve smartphone usability, which continues to evolve. In other words, the more our system develops, the more its quality (user-friendliness) would improve as regards handling complex 3D models and/or assisting surgeons.

An environment in which medical images can be easily processed and observed by linking wearable devices and sensors to smartphones or tablet computers is becoming more common these days, but we must not forget to keep abreast of related laws and guidelines. OsiriX MD has been licensed by the US Food and Drug Administration but has not been approved in Japan. It is critical that we solve these issues first to realize the clinical application of visualized images of individual patients (3D models) in surgery simulation and/or navigation.

### Conclusions

Using a smartphone, we built a simple system in which multiple people are able to observe a 3D model created by OsiriX. Although observation by multiple persons was possible, we found problems presumably caused by poor smartphone performance. Improving smartphone performance may be the key factor in establishing an inexpensive and user-friendly 3D observation environment.

## Abbreviations

2D: two-dimensional
3D: three-dimensional
AR: augmented reality
CT: computed tomography
HMD: head-mounted display
VR: virtual reality
